# Advancing science and education through a novel collaboration platform between the University of Michigan and Peking University Health Science Center

**DOI:** 10.1096/fba.2020-00128

**Published:** 2021-03-04

**Authors:** Joseph C. Kolars, Qimin Zhan

**Affiliations:** ^1^ University of Michigan Medical School Ann Arbor MI USA; ^2^ Peking University Health Science Center Beijing China

**Keywords:** biomedical research, China, international educational exchange, support of research, translational medical research

## Abstract

Research in China has been advancing over the past decade with increasing investments from government and private entities. Collaboration with Chinese investigators and those in the United States has also increased as reflected in the growth of scientific papers with Chinese authors. Collaborations are more commonly based on faculty‐to‐faculty relationships which can be challenged by institutional or governmental policies. This paper reports on an institution‐to‐ institution collaboration, the Joint Institute for Translational and Clinical Research initiated in 2010 between the University of Michigan Medical School and Peking University Health Science Center, to enable and support collaborative faculty‐initiated research. Concomitant education and training programs have also been co‐developed. Beginning in 2011, 190 proposals from faculty‐to‐faculty partnerships have been submitted from which 59 have been selected for funding. These projects have involved over 138,000 patient subjects and resulted in 86 peer‐reviewed publications to date. Pilot data has been leveraged to secure $27.3 million dollars of extramural funding outside of China. Faculty and trainee exchanges take place regularly including an annual symposium with mechanisms to link faculty who are seeking partnerships by utilizing each other's complementary strengths and resources. As the collaboration enters its second decade, both institutions believe that the model offers a unique platform to promote faculty‐initiated collaborative research. Next steps include funding studies in prioritized scientific themes, and promoting access to high‐quality cohorts to attract industry partners and to develop sustainable financial models.

## 1 INTRODUCTION

### 1.1 Opportunities

The growth in science research and publications in China has been striking. The U.S. National Institutes of Health (NIH), the largest funder of biomedical and behavioral research in the world, has noted a 253% increase in papers with a Chinese co‐author between 2009 and 2017 at a time when the overall NIH budget was flat.^1^ By this parameter, China is the leading international collaborator on NIH‐funded research. China has been increasing support for science and engineering research over the past decade and in 2017 accounted for 23% of the world's expenditure for research and development, closely rivaling the U.S. leadership at 25%.^2^ One of the largest sources of support is the National Natural Science Foundation of China (NSFC) which allocated over 31 billion RMB ($4.6 billion U.S.) in 2019.^3^ A growing number of entities have been established to advance the biomedical research in China including private companies such as the Beijing Genomics Institute.^4^ Collaborative research is being encouraged from governmental agencies in the United States and China such as joint programs between the U.S. National Science Foundation and China's NSFC.^5^ In the United States, the NIH has a U.S.–China Program for Biomedical Collaborative Research.^6^ Drivers for collaboration include access to students, trainees, research resources, and capacity building. Also, the research environment in China can be less costly than in the west.

### 1.2 Challenges

Faculty may encounter challenges when trying to engage in collaborative research between the United States and China. First, transactional barriers such as differences in language, time‐zone, and communication styles (i.e., text vs. email vs. phone call) can be more easily overcome than the cultural differences in approaches to data integrity enforcement, authorship determination, and intellectual property allocation. Untested assumptions about how things work in one place are often erroneously generalized to the partner institution. Conflict resolution is typically culturally based and can easily become problematic if not resolved directly but diplomatically with a deep understanding of how disagreements and differences are managed in different settings. Second, collaborations between individual investigators can suffer from the absence of supportive, enabling institutional leadership while collaborations only between leadership can miss investigators’ grassroots enthusiasm necessary to catalyze the innovative opportunities. Finally, governmental oversight in the United States and China can be influenced by political relationships between the countries that may present challenges for individual researchers to navigate.

With both opportunities and challenges in mind, the University of Michigan Medical School (UMMS) set out to establish a new institution‐to‐institution collaborative platform to facilitate faculty‐initiated translational and clinical research in China. The purpose of this report is to extend the original report in 2017^7^ with a special emphasis on science research.

## 2 APPROACH

### 2.1 Overall framework and governance structure

**FIGURE 1:**
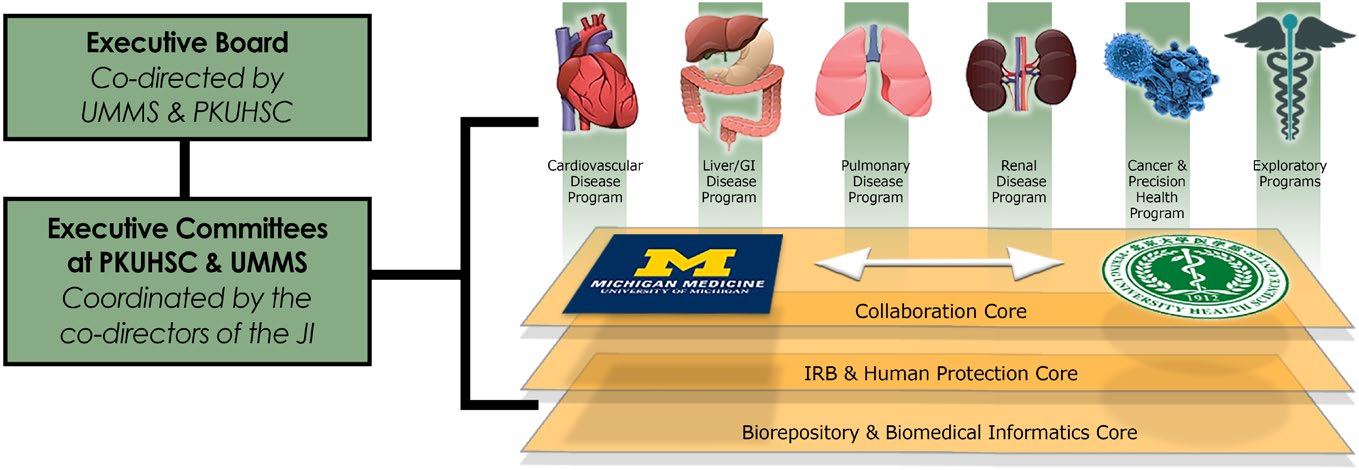
Governance structure, thematic research programs, and enabling cores of the Joint Institute (JI) for Translational and Clinical Research between Peking University Health Science Center (PKUHSC) and University of Michigan Medical School (UMMS). The Joint Institute (JI) for Translational and Clinical Research comprises six research programs: Cardiovascular Disease, Liver/GI Disease, Pulmonary Disease, Renal Disease, Cancer & Precision Health, and Exploratory. In addition, three enabling cores were established to facilitate the research, collaboration, and culture of inquiry. They are Collaboration Core, IRB & Human Protection Core, and Biorepository & Biomedical Informatics Core. The cores and research programs are overseen by the Executive Committee at each institution that are coordinated by the JI co‐directors who report to the Executive Board

As previously noted,^7^ the collaborative platform was established in 2010 as the Joint Institute for Translational and Clinical Research (JI) with the signing of a memorandum of understanding^8^ acknowledging plans to provide pilot funding for co‐investigator‐initiated studies as well as research infrastructure (i.e., three enabling cores). The cores were established to facilitate the research, collaboration, and culture of inquiry that was desired. The cores and project proposal pathways are overseen by the Executive Committee at each institution that are under the stewardship of the JI co‐directors (Joseph Kolars and Qimin Zhan) who report to the Executive Board Figure 1. An agreement outlining the governance of the JI decision‐making process, authorship, and intellectual property was defined and agreed upon by the legal offices of both universities. Furthermore, the intended goals by which success would be measured at 5 years was clearly articulated and agreed upon by both sides. The overarching goal is the generation of collaborative studies that could be leveraged for external funding with results that would improve the understanding of diseases of mutual interest.

### 2.2 Equal investment and equal partnership

Seven million dollars of funding was initially provided by each side to be spent on projects and enabling cores within their own respective institutions. Each of the enabling cores was jointly designed and managed by the faculty and staff at each institution. One of the foundational aspects of the JI is to ensure that all projects are investigator‐initiated and that all decisions are jointly agreed upon. Each project requires co‐Principal Investigators (PI) at each institution who collaboratively make decisions on developing the proposal, formulating the budget, and executing the project once funded. Funding for all projects receive equivalent monetary awards from PKUHSC and UMMS. The Executive Board and the Executive Committee consist of leadership from both institutions with a clear understanding that all decisions, including the dispersion of funds, will be jointly determined. Another defining element of the JI was ensuring that collaboration take place at the investigator level, most often working within the “thematic programs” that are co‐led by investigators from both institutions Figure 1. These thematic areas were initially chosen based on the disciplinary interests of the leadership from both institutions who were originally interested in establishing a collaborative platform. These leaders also recognized that these themes include a substantial spectrum of burden of disease for both the United States and China such that novel or collaborative solutions may have significant impact for both countries. Initially, 20% of funding in each round of proposals was set aside for exploratory areas. This now varies from round‐to‐round based on scientific merit relative to those proposals submitted in the thematic areas. Finally, each enabling core is co‐led by designated faculty with staff assistance from each institution who are separate from the faculty directing the thematic programs or the investigators on funded projects. Of the total $14 M investment, 20% was set aside to fund the cores, travel, and administrative expenses. A decision was made to not invest in buildings or jointly owned structures.

### 2.3 Three enabling cores

The Biorepository and Biomedical Informatics Core defines processes by which research data and bio‐specimens are collected, stored, and jointly utilized by establishing standardized procedures. Compliance with and adherence to government policies and regulations has been stressed.

The second enabling core, the Institutional Review Board (IRB) and Human Protection Core of the JI, facilitates IRB review and approval processes at each institution. In 2010–11, leaders from the respective institutional IRBs made reciprocal visits and held regular video‐conferences to exchange processes and familiarize each other with local rules, regulations, and practices.

The third and perhaps the most instrumental enabling core is the Collaboration Core established to enhance communication, identify opportunities (i.e., connect faculty with mutual interests), optimize the management structure and process to lead to favorable outcomes, and work through problems and barriers as they emerge. The specific work by this core includes facilitation of regular communication through video‐conferencing of co‐leadership from cores, programs, and projects; management of request for proposals process; assistance with peer review processes for submitted proposals; provision of orientation sessions to new project teams; coordination of reciprocal visits and exchanges, and organization of an annual joint symposium that alternates each fall between PKUHSC and UMMS. Following institutional guidelines and governmental regulations, the Collaboration Core has also been able to facilitate the exchange of biospecimens allowing investigators in select situations to analyze samples collected by their co‐investigators in the other country.

### 2.4 Research awards

Since January 2011, 10 calls for proposals have been issued soliciting applications for Pilot Awards (funding up to $800,000 for 2 years) or Discovery Awards (funding up to $200,000 for 2 years) jointly submitted by faculty of UMMS and PKUHSC. Prior to submitting full proposals, investigators are required to first submit a letter of intent to the Executive Committee that will consider if the concept pertains to diseases of importance to both countries and has the potential to generate pilot data that could be leveraged for external funding. In addition to the original three programmatic themes (i.e., cardiovascular, hepatology/gastroenterology, and pulmonary), renal disease was added in 2011 because of significant faculty interest at both institutions. In 2012, it was also determined that a limited amount of funding should be set aside for “exploratory areas” outside of these four main programmatic themes. In 2018, Cancer and Precision Health was added as a thematic area.

After the letter‐of‐intent review, investigators are invited to submit a full proposal, with feedback on how to optimize chances of success, which is evaluated independently by peer review boards facilitated by the Michigan Institute for Clinical and Health Research at UMMS and the Office of Research at PKUHSC where the relative merits are reviewed and constructive comments provided. Investigators then have an opportunity to respond to the comments and improve their proposals before the final ranking is determined by the internal review committees and submitted to the leadership of the Executive Committees for decisions on funding. This requires thoughtful, trusting conversations to reconcile differences in how proposals may be viewed on both sides so as to maximize the quality of the science and its potential impact.

## 3 OUTCOMES

Since the first round of request for proposals in February 2011, 190 full proposals were submitted for review of which 59 were selected for funding Table 1. To date, these JI‐funded projects have generated 86 peer‐reviewed publications that all have a translational science component (i.e., wet‐lab or computational science approaches). In addition to 17 NIH awards, extramural support includes one collaborative award with the NIH Fogarty Chronic Disease Network, and four industry awards for a total of $5.3 million in U.S. extramural funding. Three patent submissions are underway through Technology Transfer Offices at the respective institutions (targeted imaging of hepatocellular carcinoma, valproic acid for the treatment of myocardial ischemia, and CitH3 in the diagnosis and management of septic shock). Other notable accomplishments include the development of a new specific biomarker in the urine for advancing renal disease,^9^, ^10^ and a body of work on the relationship between air pollution and cardiac disease that has advanced our understanding of disease mechanisms^11^ ^,^ ^12^ and informed policy discussions at WHO.

**TABLE 1:** Summary of JI funded projects and the outcomes (April 2011 through September 2020)

Thematic programs	No. of Pilot/Discovery Awards	JI funding^b^	No. of research samples/patients^c^	No. of publications to date^d^	Extramural funding secured—UM^e^	Extramural Funding Secured—PKUHSC
Cardiovascular	9/5	$6,043,351	19,271	36	$14,628,096	$1,045,000
GI/Liver	5/4	$4,000,174	2749	19	$4,869,526	$1,500,000
Renal	2/9	$2,135,927	110,469	13	$4,916,944	$1,242,000
Pulmonary	2/5	$2,351,963	562	8	$1,580,747	$247,000
Cancer	1/1	$800,000	0	0	0	0
Exploratory^a^	2/14	$4,389,927	5907	10	$1,280,000	$429,000
Total	21/38	$19,721,342	138,958	86	$27,275,313	$4,463,000

Note

Over the past decade, the Joint Institute (JI) for Translational and Clinical Research has supported 59 projects (21 Pilot and 38 Discovery) with total awards of $19,721,342. The funded studies utilized 138,958 research samples/subjects. The JI work has generated 86 peer‐reviewed publications, and extramural funding of $27,275,313 in the United States and $4,463,000 in China.

^a^ These are areas that were not part of the five thematic programs initially identified as the priorities for the JI, and these exploratory areas include Radiology, Human Genetics, Psychiatry, Emergency Medicine, Obstetrics & Gynecology, Orthopedics, Urology, Dermatology, Dentistry, Rheumatology, Ophthalmology, Neurology, and Surgery.

^b^ Full awards are for funding up to a total of $800,000 U.S.; discovery awards are for funding up to a total of

^c^ While the cohort differs for each study, collectively 45% of the patient subjects are from the United States and 55% are from China.

^d^ See Appendix I.

^e^ Funding external to either institution secured with data or activities generated by pilot funding; only United States and international industry sources listed because all funding within China ultimately comes from the government in block allocations.

$ 200,000 U.S., with each institution contributing equally to the award; JI: Joint Institute for Translational and Clinical Research.

Return on investment for the pilot funding allocated is difficult to determine. Follow‐on funding in China is more likely to come from institutional “basket” funding from the Chinese government that can be difficult to attribute to a specific project. However, focusing on the U.S. side, extramural funding secured by the UM is $27,275,313 Table 1 from the actual expenditures of $8.2 M from pilot funding awarded to the UM investigators resulting in a return of investment of ~230%.

In almost all cases, the investigators had no prior collaborations with each other prior to those facilitated by the JI. It is also worth noting that in 2018 PKUHSC was recognized by the Chinese Ministry of Science and Technology as a National Center for International Research, with UMMS being Peking University's longest‐running and largest international partnership.

## 4 A PLATFORM FOR EDUCATION AND TRAINING

The research agendas have provided a collaborative environment for the exchange of students, trainees, staff, and faculty. Some of the exchanges, such as those that occurred with the IRB and Human Protection Core, were critical to the success of understanding UM, PKU, and international practices. Others focused on the need to transfer laboratory practices and policies that were in place at UM to partner labs in China. Of note, China relies more heavily on faculty for research work with little access to clinical research coordinators and program managers that is typical at UM, but the situation in China is improving with the support from the government. Course work and programing was developed to help foster this cohort in China while focusing on skills‐transfer to visiting learners from China. Since 2011, 107 faculty or staff from PKUHSC spent time at UMMS for short‐term (i.e., <3 months) training and 25 faculty spending time at UMMS for longer‐term experiences at times supported by competitive international awards (e.g., Milstein Medical Asian American Partnership Foundation and International Society of nephrology) In addition, a UMMS faculty member was able to successfully secure a Fulbright Award to pursue scholarship in Beijing and four UM faculty to be recognized with “Guest Professor” appointments at PKUHSC.

During this same time period, 15 students from PKUHSC participated in research experiences at UMMS in addition to five who were enrolled in a unique dual M.D/Ph.D. program. Generous philanthropic support from a donor attracted to the JI model of creating mutual benefit between UMMS and PKUHSC resulted in the creation of the Rogel Scholarship Program in 2014, which has allowed five PKUHSC medical students to enroll in the Program in Biomedical Sciences at the University of Michigan Ph.D. programs. These students will graduate with a dual M.D./Ph.D. degree from the respective institutions that optimally prepares them for future collaborative research. Thirteen students from UMMS rotated to PKUHSC for research experiences. These tend to be more short‐term rotations (several weeks) when compared to the long‐term experiences for those from PKUHSC coming to UM. There are far more PKUHSC learners who speak English and can benefit from a UM experience than there are UM learners who speak Mandarin and could benefit from longer‐term immersive experience in Beijing. In addition, learners in China have better access to governmental funding for a research experience at UM than what is currently available for U.S. students seeking longer research experiences in China.

The presence of this platform also facilitated clinical experiences for students wishing to rotate at UMMS from PKUHSC (31 to date) or for medical students/residents to rotate to PKUHSC from UMMS (25 to date). Finally, a yearly 2–3 day joint symposium that alternates between Ann Arbor, MI and Beijing, China now attracts almost 100 visitors from the partner‐institution to the host‐institution. These symposia focus on information exchange but are crucial to setting the tone for collaboration, finding new partners, and sharing best practices.

In 2017, the JI facilitated an executive education program for one of PKUHSC’s main hospitals (i.e., Peking University Third Hospital) with a focus on hospital leadership and management. Given the success of the pilot program and an increasing demand, Michigan Medicine's *Global Executive Education Program* was approved and established by the Dean's office in 2019. Three cohorts of participants were trained and certified from the program in 2019 with requests increasing from other institutions across China.

## 5 DISCUSSION

### 5.1 Lessons learned and experience gained

The JI has established a culture of collaboration by involving approximately 300 individuals, including faculty, trainees, and students from UMMS and PKUHSC to explore joint research and education opportunities. These individuals represent a majority of academic departments at each institution. Another benefit of the JI has been the ability to attract colleagues from other health science schools including nursing, public health, dentistry, and pharmacy into engagement with JI initiatives. An annual symposium regularly attracts 80–100 visitors from the partner to the host institution. Cooperation has also been carried out in the area of medical education with the exchange of students and trainees.

A number of challenges have been encountered and resolved resulting in numerous “lessons learned.” These include the need to carefully evaluate the investigators’ readiness to collaborate in addition to assessing the scientific merit of the proposals. High‐quality communication also proves to be essential including attention to format (e.g., preference for email by UMMS investigators vs. preference for phone‐calls by PKUHSC investigators), as well as optimal communication channels (e.g., more comfort among UMMS investigators in bringing problems forward to leadership relative to their PKUHSC colleagues). Being explicit regarding good communication has been essential for bridging differences in approaches to authorship determination on joint manuscripts. Finally, the ability to obtain proper government approvals for transferring genetic data from PKUHSC to UMMS requires understanding and diligence to ensure that all proper procedures are followed. Only one approved project has been discontinued because of unresolved challenges mentioned above.

Critical determinants of success include having co‐ownership at all levels with co‐investment of resources that remain in the respective countries but are mutually determined. It is essential to have a commitment to building trust, working out differences, and ensuring transparency, and a willingness to see good intentions on both sides. The engagement and participation of “boundary spanners”––individuals familiar with the language, culture, and practices of academic communities in China and the United States––has also been instrumental. Of the 10 members on our Executive Board, all from PKUHSC have had training in the United States and none of the 5 from UMMS are native mandarin speakers. Of the 53 co‐leads, co‐directors, and funded PIs, 52 from PKUHSC have had training in the United States (one at University of Michigan), and 19 out of 64 from UMMS are native mandarin speakers. Lastly, the need to define a communication plan upfront with a process for resolving difficulties that will arise cannot be over emphasized. This is in contrast to approaches that attempt to establish processes when a problem has arisen that is already altering the spirit of the relationships.

### 5.2 The value proposition of a JI structure

Collaborative science between China and the United States is well underway, typically driven by faculty who wish to work with each other making use of standard institutional pathways and policies currently in place. The value of a different, often parallel, structure to enable research should be critically evaluated. The key features of the JI that provide added value include:

- Problem solving (e.g., regarding communication, cultural differences); and
- Opportunity seeking (e.g., brokering new partnerships to help investigators find each other and develop new projects, sharing funding opportunities).

An additional value to the JI is the establishment of a continuous, trusting relationship between leadership and faculty at both institutions that can be leveraged when new issues arise outside of the research domains that could benefit from a collaborative approach. One specific example includes how leadership and investigator teams started sharing best practices in the spring of 2020 when the United States was experiencing a surge in cases from the COVID‐19 pandemic. A facilitated conference call with teams in Beijing allowed UM faculty to understand best practices to patient‐care learned in China that could be applied at the UM before published data were available.

### 5.3 Next steps

The JI has already experienced a substantial turn‐over in leadership that normally occurs in academic settings, confirming that ongoing success is not dependent on any particular leader or personality. Both institutions expressed confidence in the model by repeating investment of $7 M from each side for a second 5‐year period (2015–20). Joint discussions have resulted in an adjustment to JI strategies such that more emphasis will be placed on disease processes with cross‐cutting themes (e.g., metabolism and cancer) rather than the organ‐based approaches to building collaborations which characterized the first 10 years. In addition, prioritization will be placed on building high‐quality data repositories for partnerships with industry such that private–public partnerships can be a source for ongoing JI funding.

There is a popular Chinese saying “十年磨一剑,” which means “it takes ten years to forge a fine sword.” Over the past decade, the JI’s collaboration platform has been well established. We are optimistic regarding the long‐term success of the JI as this model has been deemed valuable by the leadership and the faculty at both institutions. The ultimate test of this model will be the ability to generate new knowledge that will contribute to the health of populations in both countries.

## AUTHOR CONTRIBUTIONS

Joseph Kolars wrote the paper; Qimin Zhan edited the paper.

## APPENDIX I

(Papers published from JI support to date, September 2020).

1. Zhou, J., Biesterveld, B. E., Li, Y., Wu, Z., Tian, Y., Williams, A. M., Tian, S., Gao, W., Bhatti, U. F., Duan, X., Wang, T., Zhang, J., Jiang, B., Wang, Z., and Alam, H. B. (2020) Peptidylarginine Deiminase 2 Knockout Improves Survival in hemorrhagic shock. Shock 54, 458‐463
2. Wu, Z., Tian, Y., Alam, H. B., Li, P., Duan, X., Williams, A. M., Liu, B., Ma, J., and Li, Y. (2020) PAD2 Mediates Caspase‐1 Associated Lethality in Pseudomonas Aeruginosa Pneumonia Induced Sepsis. J Infect Dis, published online July 30, 2020
3. Wu, Z., Deng, Q., Pan, B., Alam, H. B., Tian, Y., Bhatti, U. F., Liu, B., Mondal, S., Thompson, P. R., and Li, Y. (2020) Inhibition of PAD2 Improves Survival in a Mouse Model of Lethal LPS‐Induced Endotoxic Shock. Inflammation 43, 1436‐1445
4. Wu, L., Li, X. Q., Chang, D. Y., Zhang, H., Li, J. J., Wu, S. L., Zhang, L. X., Chen, M., and Zhao, M. H. (2020) Associations of urinary epidermal growth factor and monocyte chemotactic protein‐1 with kidney involvement in patients with diabetic kidney disease. Nephrol Dial Transplant 35, 291‐297
5. Tian, Y., Qu, S., Alam, H. B., Williams, A. M., Wu, Z., Deng, Q., Pan, B., Zhou, J., Liu, B., Duan, X., Ma, J., Mondal, S., Thompson, P. R., Stringer, K. A., Standiford, T. J., and Li, Y. (2020) Peptidylarginine deiminase 2 has potential as both a biomarker and therapeutic target of sepsis. JCI Insight 5(20), e138873
6. Sun, Y., Venugopal, J., Guo, C., Fan, Y., Li, J., Gong, Y., Chen, Y. E., Zhang, H., and Eitzman, D. T. (2020) Clopidogrel Resistance in a Murine Model of Diet‐Induced Obesity Is Mediated by the Interleukin‐1 Receptor and Overcome With DT‐678. Arterioscler Thromb Vasc Biol 40, 1533‐1542
7. Sun, P., Jia, J., Fan, F., Zhao, J., Huo, Y., Ganesh, S. K., and Zhang, Y. (2020) Hemoglobin and erythrocyte count are independently and positively associated with arterial stiffness in a community‐based study. J Hum Hypertens, published online April 7, 2020
8. Magupalli, V. G., Negro, R., Tian, Y., Hauenstein, A. V., Di Caprio, G., Skillern, W., Deng, Q., Orning, P., Alam, H. B., Maliga, Z., Sharif, H., Hu, J. J., Evavold, C. L., Kagan, J. C., Schmidt, F. I., Fitzgerald, K. A., Kirchhausen, T., Li, Y., and Wu, H. (2020) HDAC6 mediates an aggresome‐like mechanism for NLRP3 and pyrin inflammasome activation. Science 369, eaas8995
9. Lv, L., Wang, F., Wu, L., Wang, J. W., Cui, Z., Hayek, S. S., Wei, C., Reiser, J., He, K., Zhang, L., Chen, M., and Zhao, M. H. (2020) Soluble urokinase‐type plasminogen activator receptor and incident end‐stage renal disease in Chinese patients with chronic kidney disease. Nephrol Dial Transplant 35, 465‐470
10. Zhou, X. J., Klionsky, D. J., and Zhang, H. (2019) Podocytes and autophagy: a potential therapeutic target in lupus nephritis. Autophagy 15, 908‐912
11. Zhang, W., Huang, R., Wang, Y., Rao, H., Wei, L., Su, G. L., and Lok, A. S. (2019) Fat Accumulation, Liver Fibrosis, and Metabolic Abnormalities in Chinese Patients With Moderate/Severe Versus Mild Hepatic Steatosis. Hepatol Commun 3, 1585‐1597
12. Zhang, W., Chao, S., Chen, S., Rao, H., Huang, R., Wei, L., and Lok, A. S. (2019) Awareness and Knowledge of Nonalcoholic Fatty Liver Disease Among Office Employees in Beijing, China. Dig Dis Sci 64, 708‐717
13. Ye, X., Zhou, X. J., and Zhang, H. (2019) Autophagy in Immune‐Related Renal Disease. J Immunol Res 2019, 5071687
14. Wolford, B. N., Hornsby, W. E., Guo, D., Zhou, W., Lin, M., Farhat, L., McNamara, J., Driscoll, A., Wu, X., Schmidt, E. M., Norton, E. L., Mathis, M. R., Ganesh, S. K., Douville, N. J., Brummett, C. M., Kitzman, J., Chen, Y. E., Kim, K., Deeb, G. M., Patel, H., Eagle, K. A., Milewicz, D. M., C, J. W., and Yang, B. (2019) Clinical Implications of Identifying Pathogenic Variants in Individuals With Thoracic Aortic Dissection. Circ Genom Precis Med 12, e002476
15. Tian, S., Lei, I., Gao, W., Liu, L., Guo, Y., Creech, J., Herron, T. J., Xian, S., Ma, P. X., Eugene Chen, Y., Li, Y., Alam, H. B., and Wang, Z. (2019) HDAC inhibitor valproic acid protects heart function through Foxm1 pathway after acute myocardial infarction. EBioMedicine 39, 83‐94
16. Qi, Y. Y., Zhou, X. J., and Zhang, H. (2019) Autophagy and immunological aberrations in systemic lupus erythematosus. Eur J Immunol 49, 523‐533
17. Ma, J., Bao, Y. P., Wang, R. J., Su, M. F., Liu, M. X., Li, J. Q., Degenhardt, L., Farrell, M., Blow, F. C., Ilgen, M., Shi, J., and Lu, L. (2019) Effects of medication‐assisted treatment on mortality among opioids users: a systematic review and meta‐analysis. Mol Psychiatry 24, 1868‐1883
18. Liu, R., Li, H., Hua, Y., Keep, R. F., Xiao, J., Xi, G., and Huang, Y. (2019) Early Hemolysis Within Human Intracerebral Hematomas: an MRI Study. Transl Stroke Res 10, 52‐56
19. Lian, S., Chen, Q., Yao, M., Chi, C., and Fetters, M. D. (2019) Training Pathways to Working as a General Practitioner in China. Fam Med 51, 262‐270
20. Labaki, W. W., Gu, T., Murray, S., Curtis, J. L., Yeomans, L., Bowler, R. P., Barr, R. G., Comellas, A. P., Hansel, N. N., Cooper, C. B., Barjaktarevic, I., Kanner, R. E., Paine, R., 3rd, McDonald, M. N., Krishnan, J. A., Peters, S. P., Woodruff, P. G., O'Neal, W. K., Diao, W., He, B., Martinez, F. J., Standiford, T. J., Stringer, K. A., and Han, M. K. (2019) Serum amino acid concentrations and clinical outcomes in smokers: SPIROMICS metabolomics study. Sci Rep 9, 11367
21. He, Y., Wang, H., Zheng, J., Beiting, D. P., Masci, A. M., Yu, H., Liu, K., Wu, J., Curtis, J. L., Smith, B., Alekseyenko, A. V., and Obeid, J. S. (2019) OHMI: the ontology of host‐ microbiome interactions. J Biomed Semantics 10, 25
22. Feng, S., Zhou, J., Li, Z., Appelman, H. D., Zhao, L., Zhu, J., and Wang, T. D. (2019) Sorafenib encapsulated in nanocarrier functionalized with glypican‐3 specific peptide for targeted therapy of hepatocellular carcinoma. Colloids Surf B Biointerfaces 184, 110498
23. Diao, W., Labaki, W. W., Han, M. K., Yeomans, L., Sun, Y., Smiley, Z., Kim, J. H., McHugh, C., Xiang, P., Shen, N., Sun, X., Guo, C., Lu, M., Standiford, T. J., He, B., and Stringer, K. A. (2019) Disruption of histidine and energy homeostasis in chronic obstructive pulmonary disease. Int J Chron Obstruct Pulmon Dis 14, 2015‐2025
24. Chen, S., Chao, S., Konerman, M., Zhang, W., Rao, H., Wu, E., Lin, A., Wei, L., and Lok, A. S. (2019) Survey of Nonalcoholic Fatty Liver Disease Knowledge, Nutrition, and Physical Activity Patterns Among the General Public in Beijing, China. Dig Dis Sci 64, 3480‐3488
25. Canugovi, C., Stevenson, M. D., Vendrov, A. E., Hayami, T., Robidoux, J., Xiao, H., Zhang, Y. Y., Eitzman, D. T., Runge, M. S., and Madamanchi, N. R. (2019) Increased mitochondrial NADPH oxidase 4 (NOX4) expression in aging is a causative factor in aortic stiffening. Redox Biol 26, 101288
26. Ye, X., Zhou, X. J., and Zhang, H. (2018) Exploring the Role of Autophagy‐Related Gene 5 (ATG5) Yields Important Insights Into Autophagy in Autoimmune/Autoinflammatory Diseases. Front Immunol 9, 2334
27. Wu, L., Li, X. Q., Goyal, T., Eddy, S., Kretzler, M., Ju, W. J., Chen, M., and Zhao, M. H. (2018) Urinary epidermal growth factor predicts renal prognosis in antineutrophil cytoplasmic antibody‐associated vasculitis. Ann Rheum Dis 77, 1339‐1344
28. Wang, J., Wang, F., Saran, R., He, Z., Zhao, M. H., Li, Y., Zhang, L., and Bragg‐Gresham, J. (2018) Mortality risk of chronic kidney disease: A comparison between the adult populations in urban China and the United States. PLoS One 13, e0193734
29. Wang, G., Mathew, A. V., Yu, H., Li, L., He, L., Gao, W., Liu, X., Guo, Y., Byun, J., Zhang, J., Chen, Y. E., and Pennathur, S. (2018) Myeloperoxidase mediated HDL oxidation and HDL proteome changes do not contribute to dysfunctional HDL in Chinese subjects with coronary artery disease. PLoS One 13, e0193782
30. Wang, F., He, K., Wang, J., Zhao, M. H., Li, Y., Zhang, L., Saran, R., and Bragg‐Gresham, J. L. (2018) Prevalence and Risk Factors for CKD: A Comparison Between the Adult Populations in China and the United States. Kidney Int Rep 3, 1135‐1143
31. Tripathi, P., Deng, F., Scruggs, A. M., Chen, Y., and Huang, S. K. (2018) Variation in doses and duration of particulate matter exposure in bronchial epithelial cells results in upregulation of different genes associated with airway disorders. Toxicol In Vitro 51, 95‐105
32. Takemoto, Y., Slough, D. P., Meinke, G., Katnik, C., Graziano, Z. A., Chidipi, B., Reiser, M., Alhadidy, M. M., Ramirez, R., Salvador‐Montanes, O., Ennis, S., Guerrero‐Serna, G., Haburcak, M., Diehl, C., Cuevas, J., Jalife, J., Bohm, A., Lin, Y. S., and Noujaim, S. F. (2018) Structural basis for the antiarrhythmic blockade of a potassium channel with a small molecule. FASEB J 32, 1778‐1793
33. Qi, Y. Y., Zhou, X. J., Cheng, F. J., Hou, P., Ren, Y. L., Wang, S. X., Zhao, M. H., Yang, L., Martinez, J., and Zhang, H. (2018) Increased autophagy is cytoprotective against podocyte injury induced by antibody and interferon‐alpha in lupus nephritis. Ann Rheum Dis 77, 1799‐ 1809
34. Mathew, A. V., Yu, J., Guo, Y., Byun, J., Chen, Y. E., Wang, L., Liu, M., Bard, R. L., Morishita, M., Huang, W., Li, J., Harkema, J. R., Rajagopalan, S., Pennathur, S., and Brook, R. D. (2018) Effect of Ambient Fine Particulate Matter Air Pollution and Colder Outdoor Temperatures on High‐Density Lipoprotein Function. Am J Cardiol 122, 565‐570
35. Ma, J., Li, X. D., Wang, T. Y., Li, S. X., Meng, S. Q., Blow, F. C., Ilgen, M., Degenhardt, L., Lappin, J., Wu, P., Shi, J., Bao, Y. P., and Lu, L. (2018) Relationship between the duration of methamphetamine use and psychotic symptoms: A two‐year prospective cohort study. Drug Alcohol Depend 187, 363‐369
36. Lv, L., Wang, J., Gao, B., Wu, L., Wang, F., Cui, Z., He, K., Zhang, L., Chen, M., and Zhao, M. H. (2018) Serum uromodulin and progression of kidney disease in patients with chronic kidney disease. J Transl Med 16, 316
37. Liang, Y., Pan, B., Alam, H. B., Deng, Q., Wang, Y., Chen, E., Liu, B., Tian, Y., Williams, M., Duan, X., Wang, Y., Zhang, J., and Li, Y. (2018) Inhibition of peptidylarginine deiminase alleviates LPS‐induced pulmonary dysfunction and improves survival in a mouse model of lethal endotoxemia. Eur J Pharmacol 833, 432‐440
38. Li, J., Yan, S., Liu, Z., Zhou, Y., Pan, Y., Yuan, W., Liu, M., Tan, Q., Tian, G., Dong, B., Cai, H., Wu, N., and Ke, Y. (2018) Multiregional Sequencing Reveals Genomic Alterations and Clonal Dynamics in Primary Malignant Melanoma of the Esophagus. Cancer Res 78, 338‐347
39. Huang, W., Wang, L., Li, J., Liu, M., Xu, H., Liu, S., Chen, J., Zhang, Y., Morishita, M., Bard, R. L., Harkema, J. R., Rajagopalan, S., and Brook, R. D. (2018) Short‐Term Blood Pressure Responses to Ambient Fine Particulate Matter Exposures at the Extremes of Global Air Pollution Concentrations. Am J Hypertens 31, 590‐599
40. Cao, S., Hua, Y., Keep, R. F., Chaudhary, N., and Xi, G. (2018) Minocycline Effects on Intracerebral Hemorrhage‐Induced Iron Overload in Aged Rats: Brain Iron Quantification With Magnetic Resonance Imaging. Stroke 49, 995‐1002
41. Yuan, W., Liu, Z., Lei, W., Sun, L., Yang, H., Wang, Y., Ramdas, S., Dong, X., Xu, R., Cai, H., Li, J. Z., and Ke, Y. (2017) Mutation landscape and intra‐tumor heterogeneity of two MANECs of the esophagus revealed by multi‐region sequencing. Oncotarget 8, 69610‐69621
42. Yang, Y., Liu, B., Xu, J., Wang, J., Wu, J., Shi, C., Xu, Y., Dong, J., Wang, C., Lai, W., Zhu, J., Xiong, L., Zhu, D., Li, X., Yang, W., Yamauchi, T., Sugawara, A., Li, Z., Sun, F., Li, X., Li, C., He, A., Du, Y., Wang, T., Zhao, C., Li, H., Chi, X., Zhang, H., Liu, Y., Li, C., Duo, S., Yin, M., Shen, H., Belmonte, J. C. I., and Deng, H. (2017) Derivation of Pluripotent Stem Cells with In Vivo Embryonic and Extraembryonic Potency. Cell 169, 243‐257 e225
43. Yang, M., Rao, H. Y., Feng, B., Wu, E., Wei, L., and Lok, A. S. (2017) Patient Education Improves Patient Knowledge and Acceptance on Antiviral Therapy of Hepatitis C in Rural China. Chin Med J (Engl) 130, 2750‐2751
44. Wu, E., Yang, M., Rao, H., Fu, S., Feng, B., Fei, R., Lin, A., Fontana, R. J., Wei, L., and Lok, A. S. (2017) Temporal Changes in the Modes of Hepatitis C Virus Transmission in the USA and Northern China. Dig Dis Sci 62, 2141‐2149
45. Wang, L., Meier, E. M., Tian, S., Lei, I., Liu, L., Xian, S., Lam, M. T., and Wang, Z. (2017) Transplantation of Isl1(+) cardiac progenitor cells in small intestinal submucosa improves infarcted heart function. Stem Cell Res Ther 8, 230
46. Takemoto, Y., Ramirez, R. J., Kaur, K., Salvador‐Montanes, O., Ponce‐Balbuena, D., Ramos‐Mondragon, R., Ennis, S. R., Guerrero‐Serna, G., Berenfeld, O., and Jalife, J. (2017) Eplerenone Reduces Atrial Fibrillation Burden Without Preventing Atrial Electrical Remodeling. J Am Coll Cardiol 70, 2893‐2905
47. Rao, H., Wu, E., Fu, S., Yang, M., Feng, B., Lin, A., Fei, R., Fontana, R. J., Lok, A. S., and Wei, L. (2017) The higher prevalence of truncal obesity and diabetes in American than Chinese patients with chronic hepatitis C might contribute to more rapid progression to advanced liver disease. Aliment Pharmacol Ther 46, 731‐740
48. Parikh, N. D., Fu, S., Rao, H., Yang, M., Li, Y., Powell, C., Wu, E., Lin, A., Xing, B., Wei, L., and Lok, A. S. F. (2017) Risk Assessment of Hepatocellular Carcinoma in Patients with Hepatitis C in China and the USA. Dig Dis Sci 62, 3243‐3253
49. Pan, B., Alam, H. B., Chong, W., Mobley, J., Liu, B., Deng, Q., Liang, Y., Wang, Y., Chen, E., Wang, T., Tewari, M., and Li, Y. (2017) CitH3: a reliable blood biomarker for diagnosis and treatment of endotoxic shock. Sci Rep 7, 8972
50. Lu, X., Peloso, G. M., Liu, D. J., Wu, Y., Zhang, H., Zhou, W., Li, J., Tang, C. S., Dorajoo, R., Li, H., Long, J., Guo, X., Xu, M., Spracklen, C. N., Chen, Y., Liu, X., Zhang, Y., Khor, C. C., Liu, J., Sun, L., Wang, L., Gao, Y. T., Hu, Y., Yu, K., Wang, Y., Cheung, C. Y. Y., Wang, F., Huang, J., Fan, Q., Cai, Q., Chen, S., Shi, J., Yang, X., Zhao, W., Sheu, W. H., Cherny, S. S., He, M., Feranil, A. B., Adair, L. S., Gordon‐Larsen, P., Du, S., Varma, R., Chen, Y. I., Shu, X. O., Lam, K. S. L., Wong, T. Y., Ganesh, S. K., Mo, Z., Hveem, K., Fritsche, L. G., Nielsen, J. B., Tse, H. F., Huo, Y., Cheng, C. Y., Chen, Y. E., Zheng, W., Tai, E. S., Gao, W., Lin, X., Huang, W., Abecasis, G., Consortium, G., Kathiresan, S., Mohlke, K. L., Wu, T., Sham, P. C., Gu, D., and Willer, C. J. (2017) Exome chip meta‐ analysis identifies novel loci and East Asian‐specific coding variants that contribute to lipid levels and coronary artery disease. Nat Genet 49, 1722‐1730
51. Liu, R., Cao, S., Hua, Y., Keep, R. F., Huang, Y., and Xi, G. (2017) CD163 Expression in Neurons After Experimental Intracerebral Hemorrhage. Stroke 48, 1369‐1375
52. Li, Y., Tian, S., Lei, I., Liu, L., Ma, P., and Wang, Z. (2017) Transplantation of multipotent Isl1+ cardiac progenitor cells preserves infarcted heart function in mice. Am J Transl Res 9, 1530‐1542
53. Kolars, J. C., Fang, W., Zheng, K., Huang, A. Y., Sun, Q., Wang, Y., Woolliscroft, J. O., and Ke, Y. (2017) Collaboration Platforms in China for Translational and Clinical Research: The Partnership Between Peking University Health Science Center and the University of Michigan Medical School. Acad Med 92, 370‐373
54. Ejike, C., Wang, L., Liu, M., Wang, W., Morishita, M., Bard, R. L., Huang, W., Harkema, J., Rajagopalan, S., and Brook, R. D. (2017) Personal‐level exposure to environmental temperature is a superior predictor of endothelial‐dependent vasodilatation than outdoor‐ ambient level. J Am Soc Hypertens 11, 746‐753 e741
55. Ding, F., Wickman, L., Wang, S. Q., Zhang, Y., Wang, F., Afshinnia, F., Hodgin, J., Ding, J., and Wiggins, R. C. (2017) Accelerated podocyte detachment and progressive podocyte loss from glomeruli with age in Alport Syndrome. Kidney Int 92, 1515‐1525
56. Brook, R. D., and Rajagopalan, S. (2017) "Stressed" About Air Pollution: Time for Personal Action. Circulation 136, 628‐631
57. Brook, R. D., Newby, D. E., and Rajagopalan, S. (2017) Air Pollution and Cardiometabolic Disease: An Update and Call for Clinical Trials. Am J Hypertens 31, 1‐10
58. Zhou, Q., Li, Z., Zhou, J., Joshi, B. P., Li, G., Duan, X., Kuick, R., Owens, S. R., and Wang, T. D. (2016) In vivo photoacoustic tomography of EGFR overexpressed in hepatocellular carcinoma mouse xenograft. Photoacoustics 4, 43‐54
59. Zhou, C., Chan, H. P., Dong, Q., Couriel, D. R., Pawarode, A., Hadjiiski, L. M., and Wei, J. (2016) Quantitative Analysis of MR Imaging to Assess Treatment Response for Patients with Multiple Myeloma by Using Dynamic Intensity Entropy Transformation: A Preliminary Study. Radiology 278, 449‐457
60. Zhao, T., Pan, B., Alam, H. B., Liu, B., Bronson, R. T., Deng, Q., Wu, E., and Li, Y. (2016) Protective effect of Cl‐amidine against CLP‐induced lethal septic shock in mice. Sci Rep 6, 36696
61. Yang, M., Wu, E., Rao, H., Du, F. H., Xie, A., Cheng, S., Rodd, C., Lin, A., Wei, L., and Lok, A. S. (2016) A Comparative Study of Liver Disease Care in the USA and Urban and Rural China. Dig Dis Sci 61, 2847‐2856
62. Takemoto, Y., Ramirez, R. J., Yokokawa, M., Kaur, K., Ponce‐Balbuena, D., Sinno, M. C., Willis, B. C., Ghanbari, H., Ennis, S. R., Guerrero‐Serna, G., Henzi, B. C., Latchamsetty, R., Ramos‐Mondragon, R., Musa, H., Martins, R. P., Pandit, S. V., Noujaim, S. F., Crawford, T., Jongnarangsin, K., Pelosi, F., Bogun, F., Chugh, A., Berenfeld, O., Morady, F., Oral, H., and Jalife, J. (2016) Galectin‐3 Regulates Atrial Fibrillation Remodeling and Predicts Catheter Ablation Outcomes. JACC Basic Transl Sci 1, 143‐154
63. Pandit, S. V., Anumonwo, J., and Jalife, J. (2016) Atrial Fibrillation Susceptibility in Obesity: An Excess Adiposity and Fibrosis Complicity? Circ Res 118, 1468‐1471
64. Liu, L., Lei, I., and Wang, Z. (2016) Improving cardiac reprogramming for heart regeneration. Curr Opin Organ Transplant 21, 588‐594
65. Liu, L., Lei, I., Karatas, H., Li, Y., Wang, L., Gnatovskiy, L., Dou, Y., Wang, S., Qian, L., and Wang, Z. (2016) Targeting Mll1 H3K4 methyltransferase activity to guide cardiac lineage specific reprogramming of fibroblasts. Cell Discov 2, 16036
66. Li, Z., Zhou, Q., Zhou, J., Duan, X., Zhu, J., and Wang, T. D. (2016) In vivo fluorescence imaging of hepatocellular carcinoma xenograft using near‐infrared labeled epidermal growth factor receptor (EGFR) peptide. Biomed Opt Express 7, 3163‐3169
67. Huang, J., Guan, M. L., Balch, J., Wu, E., Rao, H., Lin, A., Wei, L., and Lok, A. S. (2016) Survey of hepatitis B knowledge and stigma among chronically infected patients and uninfected persons in Beijing, China. Liver Int 36, 1595‐1603
68. Group, C. C. H. W. (2016) Meta‐analysis of rare and common exome chip variants identifies S1PR4 and other loci influencing blood cell traits. Nat Genet 48, 867‐876
69. Wu, E., Wang, T., Lin, T., Chen, X., Guan, Z., Cao, C., Rao, H., Yang, M., Feng, B., Pui, S., Chan, M., Fu, S., Lin, A., Wei, L., and Lok, A. S. (2015) A comparative study of patients' attitudes toward clinical research in the United States and urban and rural China. Clin Transl Sci 8, 123‐131
70. Wu, E., Chen, X., Guan, Z., Cao, C., Rao, H., Feng, B., Chan, M., Fu, S., Lin, A., Wei, L., and Lok, A. S. (2015) A comparative study of patients' knowledge about hepatitis C in the United States and in urban and rural China. Hepatol Int 9, 58‐66
71. Tian, S., Liu, Q., Gnatovskiy, L., Ma, P. X., and Wang, Z. (2015) Heart Regeneration with Embryonic Cardiac Progenitor Cells and Cardiac Tissue Engineering. J Stem Cell Transplant Biol 1
72. Tang, C. S., Zhang, H., Cheung, C. Y., Xu, M., Ho, J. C., Zhou, W., Cherny, S. S., Zhang, Y., Holmen, O., Au, K. W., Yu, H., Xu, L., Jia, J., Porsch, R. M., Sun, L., Xu, W., Zheng, H., Wong, L. Y., Mu, Y., Dou, J., Fong, C. H., Wang, S., Hong, X., Dong, L., Liao, Y., Wang, J., Lam, L. S., Su, X., Yan, H., Yang, M. L., Chen, J., Siu, C. W., Xie, G., Woo, Y. C., Wu, Y., Tan, K. C., Hveem, K., Cheung, B. M., Zollner, S., Xu, A., Eugene Chen, Y., Jiang, C. Q., Zhang, Y., Lam, T. H., Ganesh, S. K., Huo, Y., Sham, P. C., Lam, K. S., Willer, C. J., Tse, H. F., and Gao, W. (2015) Exome‐wide association analysis reveals novel coding sequence variants associated with lipid traits in Chinese. Nat Commun 6, 10206
73. Liu, Q., Tian, S., Zhao, C., Chen, X., Lei, I., Wang, Z., and Ma, P. X. (2015) Porous nanofibrous poly(L‐lactic acid) scaffolds supporting cardiovascular progenitor cells for cardiac tissue engineering. Acta Biomater 26, 105‐114
74. Ju, W., Nair, V., Smith, S., Zhu, L., Shedden, K., Song, P. X. K., Mariani, L. H., Eichinger, F. H., Berthier, C. C., Randolph, A., Lai, J. Y., Zhou, Y., Hawkins, J. J., Bitzer, M., Sampson, M. G., Thier, M., Solier, C., Duran‐Pacheco, G. C., Duchateau‐Nguyen, G., Essioux, L., Schott, B., Formentini, I., Magnone, M. C., Bobadilla, M., Cohen, C. D., Bagnasco, S. M., Barisoni, L., Lv, J., Zhang, H., Wang, H. Y., Brosius, F. C., Gadegbeku, C. A., Kretzler, M., Ercb, C. P. N., and Consortium, P. K.‐I. (2015) Tissue transcriptome‐driven identification of epidermal growth factor as a chronic kidney disease biomarker. Sci Transl Med 7, 316ra193
75. Guo, Y., Fan, Y., Zhang, J., Lomberk, G. A., Zhou, Z., Sun, L., Mathison, A. J., Garcia‐ Barrio, M. T., Zhang, J., Zeng, L., Li, L., Pennathur, S., Willer, C. J., Rader, D. J., Urrutia, R., and Chen, Y. E. (2015) Perhexiline activates KLF14 and reduces atherosclerosis by modulating ApoA‐I production. J Clin Invest 125, 3819‐3830
76. Chaudhary, N., Pandey, A. S., Gemmete, J. J., Hua, Y., Huang, Y., Gu, Y., and Xi, G. (2015) Diffusion tensor imaging in hemorrhagic stroke. Exp Neurol 272, 88‐96
77. Wei, L., and Lok, A. S. (2014) Impact of new hepatitis C treatments in different regions of the world. Gastroenterology 146, 1145‐1150 e1141‐1144
78. Tong, X., Lv, P., Mathew, A. V., Liu, D., Niu, C., Wang, Y., Ji, L., Li, J., Fu, Z., Pan, B., Pennathur, S., Zheng, L., and Huang, Y. (2014) The compensatory enrichment of sphingosine ‐1‐ phosphate harbored on glycated high‐density lipoprotein restores endothelial protective function in type 2 diabetes mellitus. Cardiovasc Diabetol 13, 82
79. Pandey, A. S., and Xi, G. (2014) Intracerebral hemorrhage: a multimodality approach to improving outcome. Transl Stroke Res 5, 313‐315
80. Ganesh, S. K., Chasman, D. I., Larson, M. G., Guo, X., Verwoert, G., Bis, J. C., Gu, X., Smith, A. V., Yang, M. L., Zhang, Y., Ehret, G., Rose, L. M., Hwang, S. J., Papanicolau, G. J., Sijbrands, E. J., Rice, K., Eiriksdottir, G., Pihur, V., Ridker, P. M., Vasan, R. S., Newton‐ Cheh, C., Global Blood Pressure Genetics, C., Raffel, L. J., Amin, N., Rotter, J. I., Liu, K., Launer, L. J., Xu, M., Caulfield, M., Morrison, A. C., Johnson, A. D., Vaidya, D., Dehghan, A., Li, G., Bouchard, C., Harris, T. B., Zhang, H., Boerwinkle, E., Siscovick, D. S., Gao, W., Uitterlinden, A. G., Rivadeneira, F., Hofman, A., Willer, C. J., Franco, O. H., Huo, Y., Witteman, J. C., Munroe, P. B., Gudnason, V., Palmas, W., van Duijn, C., Fornage, M., Levy, D., Psaty, B. M., and Chakravarti, A. (2014) Effects of long‐term averaging of quantitative blood pressure traits on the detection of genetic associations. Am J Hum Genet 95, 49‐65
81. Pan, B., Yu, B., Ren, H., Willard, B., Pan, L., Zu, L., Shen, X., Ma, Y., Li, X., Niu, C., Kong, J., Kang, S., Eugene Chen, Y., Pennathur, S., and Zheng, L. (2013) High‐density lipoprotein nitration and chlorination catalyzed by myeloperoxidase impair its effect of promoting endothelial repair. Free Radic Biol Med 60, 272‐281
82. Rao, H. Y., Sun, D. G., Yang, R. F., Liu, F., Wang, J., Feng, B., Wu, N., Fang, J. L., Song, G. J., Ma, H., Guo, F., Wang, J. H., Li, X. B., Jin, Q., Qin, H., Zhuang, H., and Wei, L. (2012) Outcome of hepatitis C virus infection in Chinese paid plasma donors: a 12‐19‐year cohort study. J Gastroenterol Hepatol 27, 526‐532
83. Rao, H. Y., Sun, D. G., Jiang, D., Yang, R. F., Guo, F., Wang, J. H., Liu, F., Zhang, H. Y., Zhang, H. H., Du, S. C., Jin, Q., Qin, H., Lok, A. S., and Wei, L. (2012) IL28B genetic variants and gender are associated with spontaneous clearance of hepatitis C virus infection. J Viral Hepat 19, 173‐181
84. Pan, B., Ma, Y., Ren, H., He, Y., Wang, Y., Lv, X., Liu, D., Ji, L., Yu, B., Wang, Y., Chen, Y. E., Pennathur, S., Smith, J. D., Liu, G., and Zheng, L. (2012) Diabetic HDL is dysfunctional in stimulating endothelial cell migration and proliferation due to down regulation of SR‐BI expression. PLoS One 7, e48530
85. Erb‐Downward, J. R., Sadighi Akha, A. A., Wang, J., Shen, N., He, B., Martinez, F. J., Gyetko, M. R., Curtis, J. L., and Huffnagle, G. B. (2012) Use of direct gradient analysis to uncover biological hypotheses in 16s survey data and beyond. Sci Rep 2, 774
86. Liu, D., Ji, L., Tong, X., Pan, B., Han, J. Y., Huang, Y., Chen, Y. E., Pennathur, S., Zhang, Y., and Zheng, L. (2011) Human apolipoprotein A‐I induces cyclooxygenase‐2 expression and prostaglandin I‐2 release in endothelial cells through ATP‐binding cassette transporter A1. Am J Physiol Cell Physiol 301, C739‐748
